# m^1^A demethylase Alkbh3 regulates neurogenesis through m^1^A demethylation of Mmp15 mRNA

**DOI:** 10.1186/s13578-024-01275-9

**Published:** 2024-07-14

**Authors:** Huan Wang, Linjie Xie, Haomin Guo, Lishi Li, Shuwei Chen, Ye Fan, Jingyuan Tian, Liping Xu, Xuejian Kong, Aiguo Xuan

**Affiliations:** 1 Department of Neurology, Institute of Neuroscience, Key Laboratory of Neurogenetics and Channelopathies of Guangdong Province and the Ministry of Education of China, The Second Affiliated Hospital of Guangzhou Medical University, Guangzhou, 510260 China; 2 School of Basic Medical Sciences, Guangzhou Medical University, Guangzhou, 511436 China; 3 Scientific Research Center of Guangzhou Medical University, Guangzhou, 511436 China; 4 The Affiliated TCM Hospital of Guangzhou Medical University, Guangzhou, 510645 China; 5 Qingyuan People’s Hospital, The Sixth Affiliated Hospital of Guangzhou Medical University, Qingyuan, 511518 China

**Keywords:** Neurogenesis, N^1^-Methyladenosine, Alkbh3, Mmp15

## Abstract

**Background:**

N^1^-Methyladenosine (m^1^A) is an abundant modification of transcripts regulating mRNA structure and translation efficiency. However, the characteristics and biological functions of mRNA m^1^A modification in adult hippocampal neurogenesis remain enigmatic.

**Results:**

We found that m^1^A demethylase Alkbh3 was dramatically enriched in neurons and neuronal genesis. Functionally, depletion of Alkbh3 in neural stem cells (NSCs) significantly decreased m^1^A modification, neuronal differentiation and proliferation coupling with increasing gliogenesis, whereas overexpressing Alkbh3 facilitated neuronal differentiation and proliferation. Mechanistically, the m^1^A demethylation of Mmp15 mRNA by Alkbh3 improved its RNA stability and translational efficacy, which promoted neurogenesis. Therapeutically, the silencing of Alkbh3 reduced hippocampal neurogenesis and impaired spatial memory in the adult mice.

**Conclusions:**

We reveal a novel function of m^1^A demethylation on Mmp15 mRNA in Alkbh3-mediated neurogenesis, which shed light on advancing Alkbh3 regulation of neurogenesis as a novel neurotherapeutic strategy.

**Supplementary Information:**

The online version contains supplementary material available at 10.1186/s13578-024-01275-9.

## Introduction

Adult hippocampal neurogenesis (AHN) generates newborn neurons throughout life in mammals and is involved in crucial hippocampal-related functions including cognition and memory [1]. AHN is heavily affected by chemogenetic and pharmacological manipulation, physiological and pathological conditions, and the local environment [2, 3]. Importantly, AHN is impaired in many neurodegenerative and neuropsychiatric disorders [4]. However, current treatments are still unable to achieve satisfactory therapeutic and preventive effects for these diseases. Thus, in-depth molecular mechanisms investigation underlying AHN becomes particularly crucial when utilizing AHN as a therapeutic target for these neurological disorders.

N1-methyladenosine (m^1^A) is a prevalent and reversible post-transcriptional RNA modification occurring in tRNA, rRNA and mRNA [5]. Dynamic m^1^A methylation in mRNA catalyzed by the writers Trmt6/Trmt61A and the eraser Alkbh3 elaborately modulates the stability and translation efficiency of mRNAs via blocking the Watson-Crick interface [6]. Dysregulation of m^1^A in mRNAs is linked with human diseases such as tumorigenesis, cardiovascular diseases, pulmonary diseases and neurological disorders [7, 8]. Alkbh3-dependent m^1^A demethylation of mRNA affects multiple biological processes and diverse cellular functions. For instance, Alkbh3 has been shown to inhibit ciliogenesis and cilia-associated developmental events in vertebrates [9]. Alkbh3 overexpression alleviates neurodegeneration and induces extension of the lifespan in C. elegans [10]. Alkbh3 is dispensable for hematopoietic stem cells (HSCs) maintenance and differentiation, but overexpression of Alkbh3 rectifies the differentiation skewing of aged HSCs [11]. Nevertheless, the features and biological functions of Alkbh3-mediated m^1^A demethylation within mRNA in neurogenesis remain perplexing.

Here we revealed that m^1^A and its demethylase Alkbh3 were significantly abundant in neurons and promoted neurogenesis both in vitro and in vivo. Moreover, Alkbh3-mediated m^1^A demethylation within mRNA enhanced the stability and translational efficacy of Mmp15 mRNA. Importantly, the silencing of Alkbh3 exhibited notably reduced hippocampal neurogenesis and behavioral defects in mice. Collectively, our results clarify the biological function and the underlying molecular mechanism of m^1^A modification demethylated by Alkbh3 in hippocampal neurogenesis, thereby providing the novel therapeutic target for hippocampal neurogenesis-related diseases.

## Methods and materials

### Cell culture and transfection

Ault hippocampal neural stem cells (NSCs) were purchased from Merck-Millipore (SCR022). The cells proliferated in neural stem cell base medium (SCM003, Millipore) containing fibroblast growth factor 2 (FGF2, 20 ng/mL, Millipore) on the dishes coated with 50 µg/ml poly-L-ornithine and 10 µg/ml fibronectin (Sigma-Aldrich). Adding 1 µM retinoic acid (Sigma-Aldrich) plus 5 µM forskolin (Sigma-Aldrich) into base medium to induce neuron-specific differentiation of NSCs, and adding 50 ng/ml LIF (Merck Millipore) plus 50 ng/ml BMP-2 (R&D Systems) into base medium to induce astrocyte-specific differentiation of NSCs. They all need to be cultivated for seven days.

The experimental plasmid construction and lentivirus packaging were provided by Shanghai Genechem Co., Ltd. These were the target sequences of short hairpin RNAs (shRNAs): Sh-Alkbh3, 5′-TGAAGATACCATTGGATCA-3′; Sh-Alkbh3, 5′-CAACCAAGACTTACAGCAT-3′, Sh-Mmp15, 5′-GCACTGACCTGCATGGAATCA-3′, Sh-Mmp15, 5′-GGATGGACACTGACAACTTCC-3′. For Alkbh3 overexpression, the sequences encoding Alkbh3 was inserted into the lentivirus which consisted of Ubi-MCS-SV40-EGFP-IRES-puro-GV367 vector. RISPR/Cas9 system was used into lentivirus vector expressing Alkbh3, designing single guide RNAs (sgRNAs) whose sequence was GCAACTACCATCTGACCTTCA. After the preparation of all lentiviruses, the optimal virus concentration of transfected cells was determined by pre-experiment. Lentivirus transfection and puromycin (1 µg/ml) screening generated stable knockdown and overexpression lines.

### Animals and virus injection

The experimental protocol was approved by the Animal Ethics Committee of Guangzhou Medical University, and the experiment was conducted in accordance with the Guidelines for Animal Protection and Use in China. Wild-type male C57BL/6 mice were pre-adapted to the experimental environment for a week, and were divided into two groups by random number assignment, namely the experimental group and the control group, with eight mice in each group. Alkbh3 retrovirus was injected into the experimental group and vector retrovirus into the control group using brain stereotaxic apparatus. Behavioral experiment and BrdU proliferation experiment could be performed three weeks later.

Alkbh3-specific shRNA (Target Seq1 TGAAGATACCATTGGATCA, Target Seq2 GCCATGAAACACCTTCCTAAT) retroviral particles were obtained from Shanghai Genechem Co., Ltd. 1 µl of retroviral solution with a titer of 2☓10^8^ units/ml was injected at a rate of 0.1 µl/min into the bilateral hippocampus (2.0 mm posterior to the bregma, ± 1.5 mm lateral to the midline, 2.0 mm deep from the top of the skull). After viral injections, the needle was retained for 20 min, and then slowly extracted. The scalps of mice were sutured, marked accordingly, and put back into the cage for follow-up experiments.

### Immunohistochemistry and immunofluorescence

For immunohistochemistry, mice brain tissues were fixed with 4% paraformaldehyde, then the brain was dehydrated with 30% sucrose, embedded with OCT compound (SAKURA), and sectioned to 30 μm in freezing microtome before treating with antigen retrieval solution (P0090, Beyotime). For immunofluorescence, the cells spread on the well plate were fixed with 4% paraformaldehyde. The experiments in vitro were independently repeated three times. The following antibodies were used in stained: anti-Alkbh3 (1:400; Novus; NBP2-55419), anti-Mmp15 (1:200; MyBioSource; MBS1753732), anti-GFAP (1:200; Abcam; ab4674), anti-Nestin (1:250; Proteintech; 66259-1-Ig), anti-beta III Tubulin (1:200; Abcam; ab78078), anti-NeuN (1:200; Abcam; ab104224), anti-BrdU (1:200; CST; 5292 S), anti-Doublecortin (1:400; CST; 4604 S).

### 5-Bromo-2′-deoxyuridine and 5-ethynyl-2′-deoxyuridine labeling

Mice in both groups were intraperitoneally injected with BrdU (Sigma) solution (dosage per 100 mg/kg of mouse body weight) for seven days at a fixed time every day, and the brain could be injected from the eighth day. During BrdU staining, slices were soaked in 2 N HCl at 37℃ for 30 min, then 0.1 M borate buffer was added at pH 8.5 and soaked at room temperature for 10 min. The other steps are basically the same as immunofluorescence. For EdU labeling, cells were treated with kFluor488-EdU method cell proliferation assay kit (KGA331, KeyGEN BioTECH) according to the manufacturer’s protocols. The experiment was performed in triplicate.

### Quantitative real-time RT-PCR

Total RNA was extracted from the cells using TRIzol reagent (Invitrogen), followed by reverse transcription using RT reagent kit with gDNA eraser (Takara, RR047A) and real-time PCR analysis using TB Green Premix Ex Taq II (Takara, RR820A). The experiment was performed in triplicate. The primer sequences were as follows: Alkbh3 forward: 5′-TCCAGAGAACGGAGAGTAA-3′: Alkbh3 reverse: 5′-GAGGAATAGGACCTGAGAAG-3′: Mmp15 forward: 5′-GCCTGCCTGGGAACATTAGT-3′: Mmp15 reverse: 5′-ATTGAAGCGCCAGTACCTGT-3′: GAPDH forward: 5′-GCGAGATCCCGCTAACATCA-3′; GAPDH reverse: 5′-CTCGTGGTTCACACCCATCA-3′.

### Western blotting

RIPA lysis buffer containing protease inhibitor (KGP702, KeyGEN BioTECH) was added to the cells in the petri dish to recapture the cells, and then collected into Eppendorf tubes, which were crushed on ice by ultrasound and centrifuged to extract the supernatant. According to the molecular weight of the target protein and the corresponding instructions, SDS-PAGE gel configuration kit was used to prepare the concentrated glue and the separation glue of the corresponding concentration. Western blotting was performed as standard protocol. The following antibodies were used in Western blotting: anti-Alkbh3 (1:800; Proteintech; 12292-1-AP), anti-Mmp15 (1:500; Bioworld ; BS7041), anti-β-actin (1:2000; CST; 4970 S), anti-rabbit HRP-linked IgG (1:2000; CST; 7074 S), anti-mouse HRP-linked IgG (1:2000; CST; 7076 S). The experiment was performed in triplicate.

### m^1^A dot blot assay

Total RNA was isolated from petri dishes with the same number of cells using Trizol reagent (Invitrogen). mRNA purification kits (Invitrogen, 61,006) purify mRNA from total RNA. Purified mRNA of the same quality was uniformly spotted on a Nitrocellulose membrane (GE Healthcare; RPN308B). After cross-linking with 254 nm UV, the membrane was blocked with 5% nonfat milk in TBST and then incubated with m^1^A specific antibody (1:500; abcam; ab208196) at 4℃ overnight. The experiment was performed in triplicate.

### m^1^A-MeRIP-seq and data process

The m^1^A-IP-Seq service was provided by Supin (Shanghai) Biotechnology Co., LTD. The cultured cells were collected and total RNA was extracted from NSCs group, astrocyte differentiation group and neuronal differentiation group, respectively. After the total RNA samples were inspected and quantified by agarose electrophoresis and Nanodrop, the mRNA was enriched with oligo (dT) magnetic beads (if the RNA was degraded or prokaryotic, it was directly treated with rRNA removal kit). All RNA sequencing libraries are completed by the kit, including the first strand cDNA generated by random primers after RNA fragmentation, the second strand cDNA synthesized by adding dUTP, the double-stranded cDNA end repair, adding A, connecting with Illumina matching splitter, and PCR amplification to obtain the final library: The constructed library was inspected by Agilent 2100, quantified by qPCR, and sequenced by Illumina NovaSea 6000 sequencer. Solexa pipe line v1.8 (Off-Line Base Caller software, v1.8) was used to process the sequencing images and obtain the sequence data. The original sequence was inspected with Pastac (vo.11.7) and filtered with Trimmomatie (vo.32). The filtered high-quality data were compared to the reference genome in the Ensenbl database (FISAT2 v210), and then exomePeak (v2.13.2) was used to determine the peaks of each sample and the differential methylation peaks of each contrast. Annotate peaks according to the annotation information in the Ensembl database and count the various transcription areas. Whether there is a peak in this paper, the motif analysis of peaks is carried out at last.

### RNA stability assay

NSCs were transfected with shRNA-Alkbh3, or Overexpression-Alkbh3 and then were incubated with 5 mM actinomycin D (Sigma) for inhibition of mRNA transcription. Samples were collected at 0, 3, 6 h post treatment, and total RNA was extracted and analyzed by qRT-PCR. The experiment was performed in triplicate.

### Polysome profiling

The translation efficiency of mRNA was detected by polysome profiling. In brief, the cells were incubated with 100 µg/ml of actinomycin D for 15 min before collection, followed by adding lysis buffer. Subsequently, the centrifuged lysate was added to a prepared 10–50% sucrose gradient and centrifuged at 36 000 rpm for 3 h, followed by separation with the gradient density separator. The sucrose gradient was then fractionated and UV absorption at 260 nm was recorded. The total and polysomal RNA fractions were extracted from each fraction and the relative expression of Mmp15 mRNA on the polysome fraction was detected by QRT-PCR. The experiment was performed in triplicate.

### Dual-luciferase reporter assay

The Dual-luciferase assay was performed based on the Dual-luciferase reporter assay system (Promega). Cells were seeded into the 24-well plates and then were transfected with the psiCHECK-2 vector (Promega) and various constructs containing the seed sequence or mutant seed sequence of Mmp15 mRNA 3′UTR. The lysates were collected and used to measure the luciferase reporter activity. The relative luciferase activities were measured by a SYNERGY microplate reader (BioTek). The experiment was performed in triplicate.

### Morris water maze

Morris water maze (MWM) experiment is a kind of experiment that forces experimental animals to swim and learn to find hidden platforms in water, mainly used to test the learning and memory ability of experimental animals to sense spatial position and sense of direction (spatial orientation). Concealed platform experiment: The pool diameter is 120 cm, the height is 50 cm, the water depth is 30 cm, and the water temperature is maintained at 26 ± 1 ℃; Four equidistant points N, E, S and W are marked on the wall of the pool as the starting point of the test. The water distribution pool is divided into four quadrants (NW, WS, SE and EN), and the platform is placed in the center of the quadrant (the distance between the platform and the center of the pool wall is equal). The platform is colorless and transparent, 10 cm in diameter and 1 cm underwater. Mice were placed in the MWM and faced the walls of the maze. The time limit for each test was 1 min. Each animal was tested a total of four times per day in each of the four quadrants, and about 5–6 days (20–24 trials) is usually enough for mice (in a 120 cm maze) to achieve stable performance. Space exploration experiments: The purpose of space exploration experiments is to determine whether the animal remembers the location of the platform. The indicators reflecting this memory include the retention time in the original platform quadrant, the number of crossing the desired platform, the average distance to the original target location, the incubation period of the first crossing the original target location, etc. The space exploration experiment should be tested at least 24 h after the end of the hidden platform experiment. All of the behavioral parameters of the mice were tracked, recorded, and analyzed using Ethovision XT 14.0 software (Noldus).

### Statistical analysis

All data were analyzed using statistical software SPSS16.0 and GraphPad Prism9.1.1. The unpaired student’s t-test was used to determine the difference between the two groups. One-way ANOVA analysis and Bonferroni multiple comparison test were used to determine the difference between the multiple groups. Escape latencies during spatial learning in the Morris water maze were analyzed via two-way ANOVA. A *P* value of 0.05 was used as the threshold for statistical significance.

## Results

### m^1^A demethylase Alkbh3 shows preferential expression in neurons

**Fig. 1:**
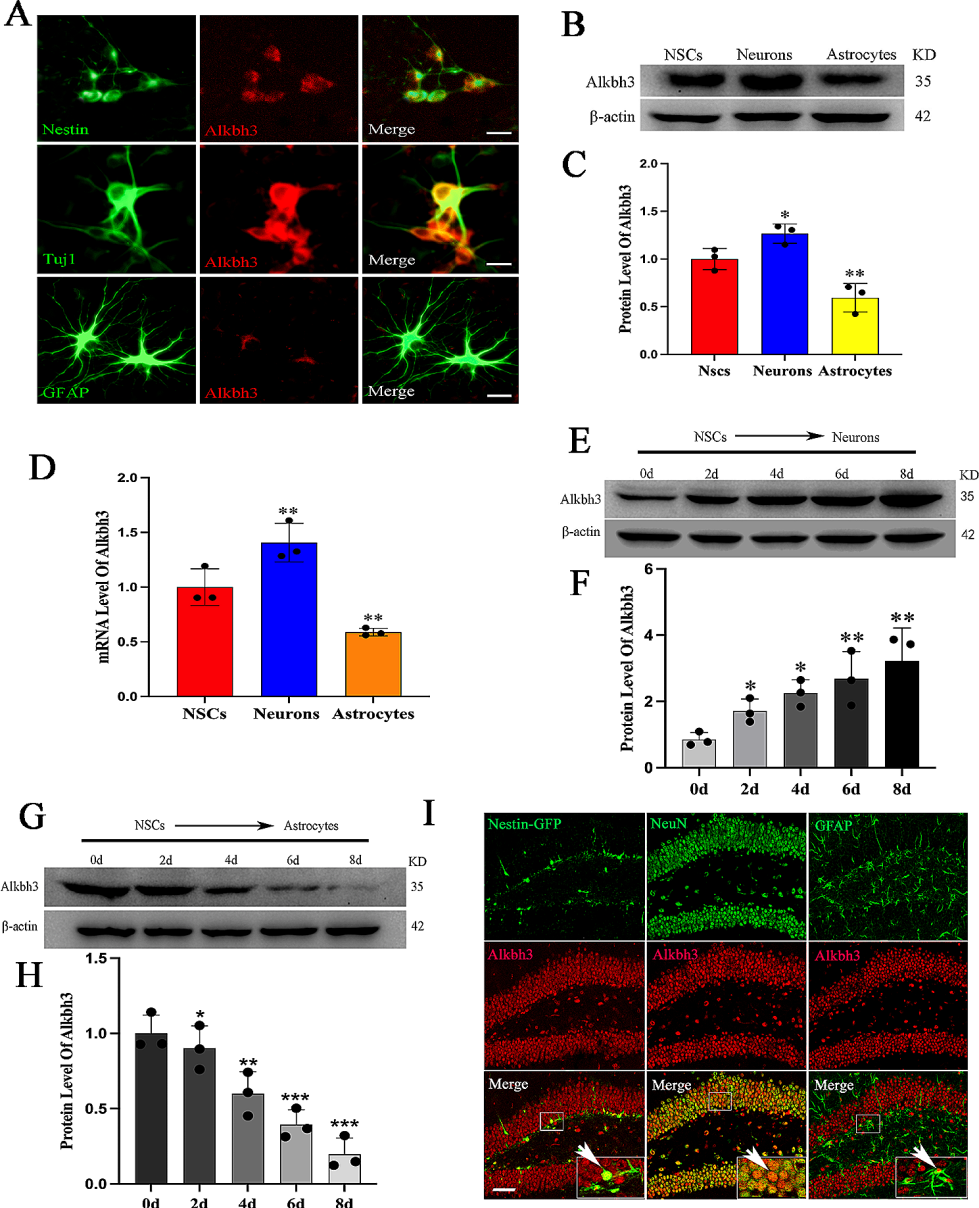
Alkbh3 demonstrates differential expression during neurogenesis (**A**) Immunofluorescence staining for Alkbh3 in Nestin + NSCs, Tuj1 + neurons and GFAP + astrocytes. Scale bar, 20 μm. (**B**,** C**) Western blotting and quantification for Alkbh3 in NSCs, neurons and astrocytes. *n* = 3, *P < 0.05, **P < 0.01 compared with NSCs. (**D**) The expressions of Alkbh3 mRNA in NSCs, neurons and astrocytes. *n* = 3, **P < 0.01 compared with NSCs. (**E**,** F**) Representative blots and quantification of Alkbh3 in neuronal differentiation of NSCs at different times. *n* = 3, *P < 0.05, **P < 0.01 compared with 0 d. (**G**,** H**) Representative blots and quantification of Alkbh3 in astrocytic differentiation of NSCs at different times. *n* = 3, *P < 0.05, **P < 0.01, ***P < 0.001 compared with 0 d. (**I**) Immunofluorescence staining for Alkbh3 costaining with Nestin, NeuN and GFAP in dentate gyrus of hippocampus. Scale bar, 50 μm. Data are represented as the mean ± SEM. NSCs, neural stem cells; Tuj1, β-tubulin; GFAP, glial fibrillary acidic protein; NeuN, neuronal nuclei; GFP, green fluorescent protein; neurons, neuronal differentiation; astrocytes, astrocyte differentiation; n represents number of independent experiments

To determine the function of m^1^A demethylase Alkbh3 during neurogenesis, we first performed immunolabeling of Alkbh3 in NSCs, neurons and astrocytes derived from NSCs. We observed significant Alkbh3 abundance in neurons but scarcity in astrocytes compared with NSCs (Fig. 1A). Western blotting and RT-qPCR analyses showed that Alkbh3 level was higher in neuronal differentiation but lower in astrocyte differentiation, in consistent with immunostaining data (Fig. 1B-H), implying that m^1^A demethylase Alkbh3 is closely related to neurogenesis. To further validate the in vitro results, the m^1^A demethylase Alkbh3 was co-stained with NSCs, neurons and astrocytes in mouse hippocampus. Interestingly, we observed that Alkbh3 immunoreactivity was markedly increased in the nucleus of NSCs and neurons (Fig. 1I). These findings indicate a crucial role of m^1^A demethylase Alkbh3 in neurogenesis.

### Alkbh3 promotes neuronal differentiation and proliferation of NSCs

**Fig. 2:**
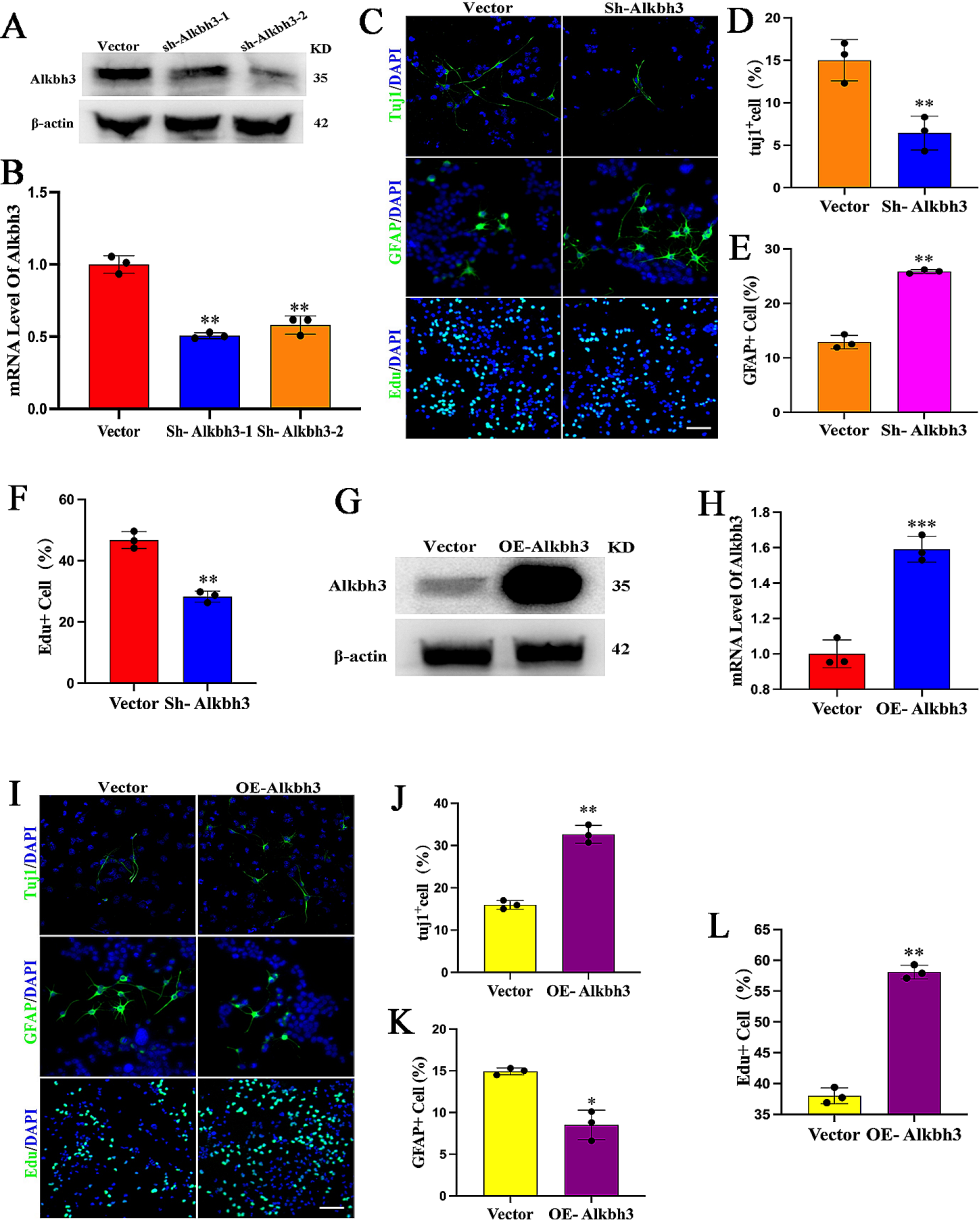
Alkbh3 facilitates neuronal differentiation (**A**,** B**) Western blot and qRT-PCR analysis confirming expression of Alkbh3 in NSCs with Alkbh3 knockdown. *n* = 3, **P < 0.01 compared with vector. (**C-F**) Immunofluorescence staining and quantification of Tuj1, GFAP and EdU in NSCs with control or Alkbh3 shRNA. Scale bar, 100 μm. *n* = 3, ***P < 0.001, **P < 0.01 compared with control. (**G**,** H**) Western blot and qRT-PCR of Alkbh3 overexpression in NSCs. *n* = 3, ***P < 0.001 compared with vector. (**I-L**) Immunofluorescence staining and quantification of Tuj1, GFAP and EdU in NSCs with control or Alkbh3 overexpression. Scale bar, 100 μm. *n* = 3, *P < 0.05, **P < 0.01 compared with vector. Data are represented as the mean ± SEM. NSCs, neural stem cells; Tuj1, β-tubulin; GFAP, glial fibrillary acidic protein; EdU, 5-ethynyl-2′-deoxyuridine; Sh-Alkbh3, shRNA Alkbh3; OE-Alkbh3, overexpressing Alkbh3; n represents number of independent experiments

To determine whether Alkbh3 is involved in the regulation of stemness and differentiation, we knocked down Alkbh3 (Fig. 2A, B) in NSCs and found that Alkbh3 depletion significantly inhibited neuronal differentiation and NSCs proliferation while enhanced glial cell differentiation (Fig. 2C-F). Conversely, forced expression of Alkbh3 promoted neuronal differentiation and NSCs proliferation (Fig. 2G-L). These results reveal the induction of neurogenesis and suppression of astrocytogenesis by m^1^A demethylase Alkbh3.

### Alkbh3 catalyzes demethylation of m^1^A in neurogenesis

**Fig. 3:**
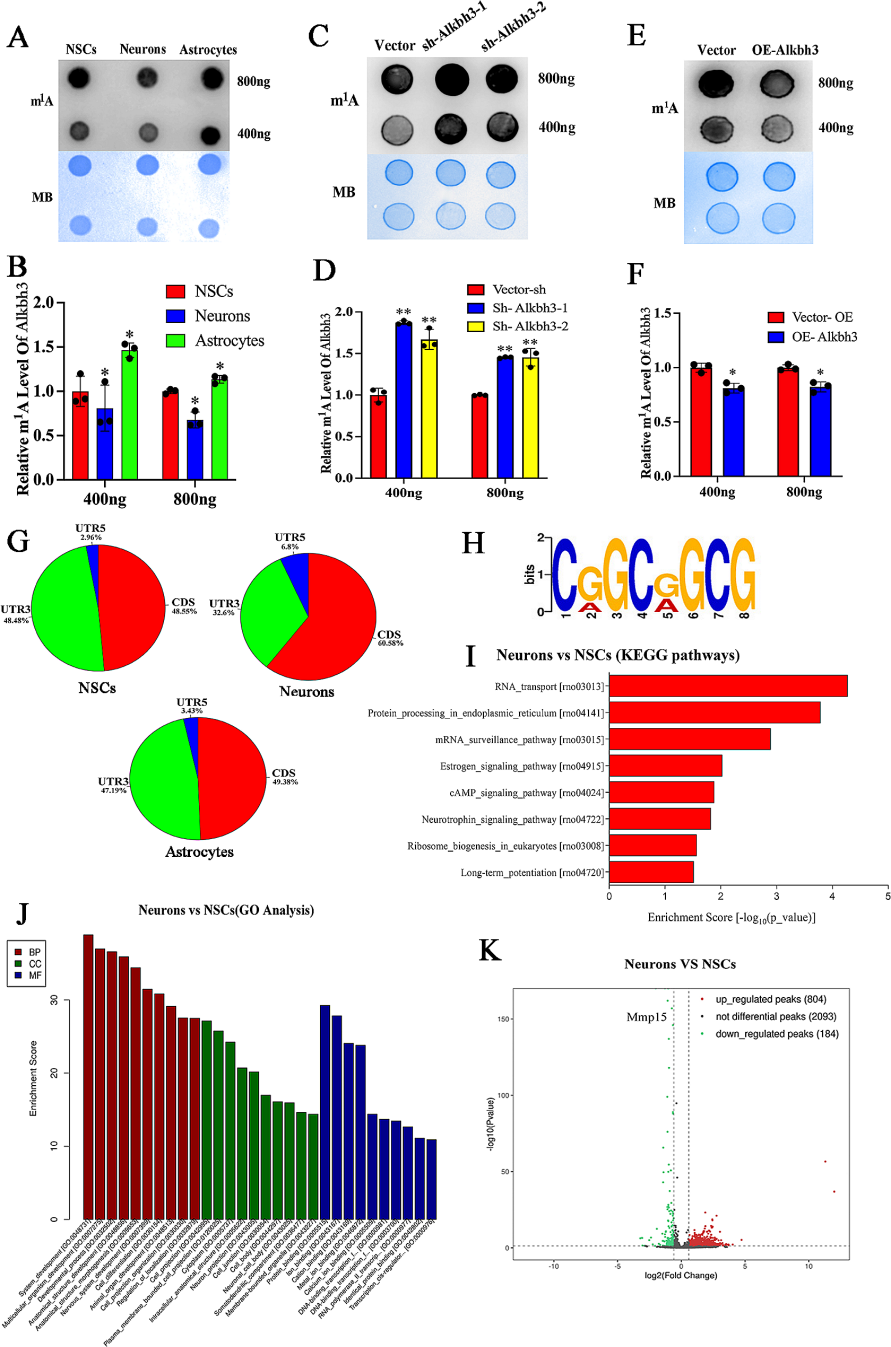
Alkbh3 regulates the transcriptome of m^1^ A-modified mRNA during neurogenesis (**A**,** B**) RNA dot blot analysis of m^1^A levels in NSCs, astrocytes and neurons. *n* = 3, *P < 0.05 compared with NSCs. (**C**,** D**) RNA dot blot analysis of m^1^A levels in NSCs treated with Alkbh3 knockdown. *n* = 3, **P < 0.01 compared with NSCs. (**E**,** F**) RNA dot blot analysis of m^1^A levels in NSCs treated with Alkbh3 overexpression. *n* = 3, *P < 0.05 compared with NSCs. (**G**) Pie charts showing the m^1^A peak distribution in different RNA regions (CDS, 5’UTR and 3’UTR) in NSCs, neurons and astrocytes. (**H**) Representative analysis of the modified region of the m^1^A transcript. (**I**,** J**) KEGG analysis and GO analysis of the m^1^A-modified genes for NSCs and differentiated neurons mRNAs. (**K**) Volcano plot showing significantly altered mRNA m^1^A peaks between NSCs and neurons. NSCs, neural stem cells; Neu, neurons; Ast, astrocytes. Data are represented as the mean ± SEM. NSCs, neural stem cells; Sh-Alkbh3, shRNA Alkbh3; OE-Alkbh3, overexpressing Alkbh3; n represents number of independent experiments. BB, biological process; CC, cellular component; MF, molecular function; GO, Gene ontology; KEGG, kyoto encyclopedia of genes and genomes; neurons, neuronal differentiation; astrocytes, astrocyte differentiation

Since Alkbh3 has been identified as the only known mRNA m^1^A demethylase, we next investigated whether Alkbh3 induces m^1^A demethylation during neurogenesis. We found a remarkable decrease m^1^A modification in neuronal differentiation and significant increase in astrocyte differentiation compared with NSCs (Fig. 3A, B), indicating a crucial role of m^1^A modification in cell fate decisions. Moreover, Alkbh3 depletion significantly increased m^1^A level compared to the control (Fig. 3C, D). Conversely, overexpression of Alkbh3 obviously decreased m^1^A level (Fig. 3E, F), indicating Alkbh3-dependent m^1^A demethylation in NSCs. Together, these results establish a clear link between Alkbh3 and m^1^A level during neurogenesis.

### Multiomics integrative analysis of m^1^A modification during neurogenesis

m^1^A methylation demonstrates potential function in regulating neurogenesis. To investigate the molecular mechanism of m^1^A mRNA modification in neurogenesis, we performed m^1^A meRIP-seq on mRNAs from NSCs, neurons and astrocytes to analyze the dynamic profiles of mRNA m^1^A methylome and observed that m^1^A peaks are enriched at CDS, 3′UTR and 5′UTR (Fig. 3G). Then, a motif analysis of mRNA m^1^A from three types of cells has a conserved sequence pattern (Fig. 3H). Collectively, these results suggest successful identification of the specific m^1^A sites in neurogenesis.

We next analyzed direct m^1^A targets in neurogenesis via m^1^A mRNA MeRIP-Seq in NSCs compared with neuronal differentiation. KEGG pathway analysis showed that these m^1^A methylation peaks were enriched in RNA transport, protein processing and neurotrophin signaling pathway (Fig. 3I). Gene ontology (GO) analysis showed that m^1^A-modified mRNAs were concentrated in genes related to neurogenesis, such as nervous system development, neuron projection and developmental process (Fig. 3J). Additionally, KEGG pathway analysis revealed m^1^A-tagged mRNA to be associated with the ribosome, proteasome and pentose phosphate pathway in NSCs compared with astrocyte differentiation (Additional file 1: Fig. S1A). GO analysis the m^1^A-tagged mRNA showed an enrichment of genes related to negative regulation of cellular process and nerve development (Additional file 1: Fig. S1B). Motif searching identified the consensus conserved sequence (Additional file 1: Fig. S1C). Of all m^1^A peaks, 804 were upregulated, while 184 were downregulated in neuronal differentiation compared to NSCs (Fig. 3K). In addition, 343 upregulated and 394 downregulated peaks were observed in astrocyte differentiation compared to NSCs (Additional file 1: Fig. S1D). Taken together, these data suggest the dynamic and diverse m^1^A changes in neurogenesis.

### m^1^A demethylation of Mmp15 enhances its RNA stability and translational efficacy

**Fig. 4:**
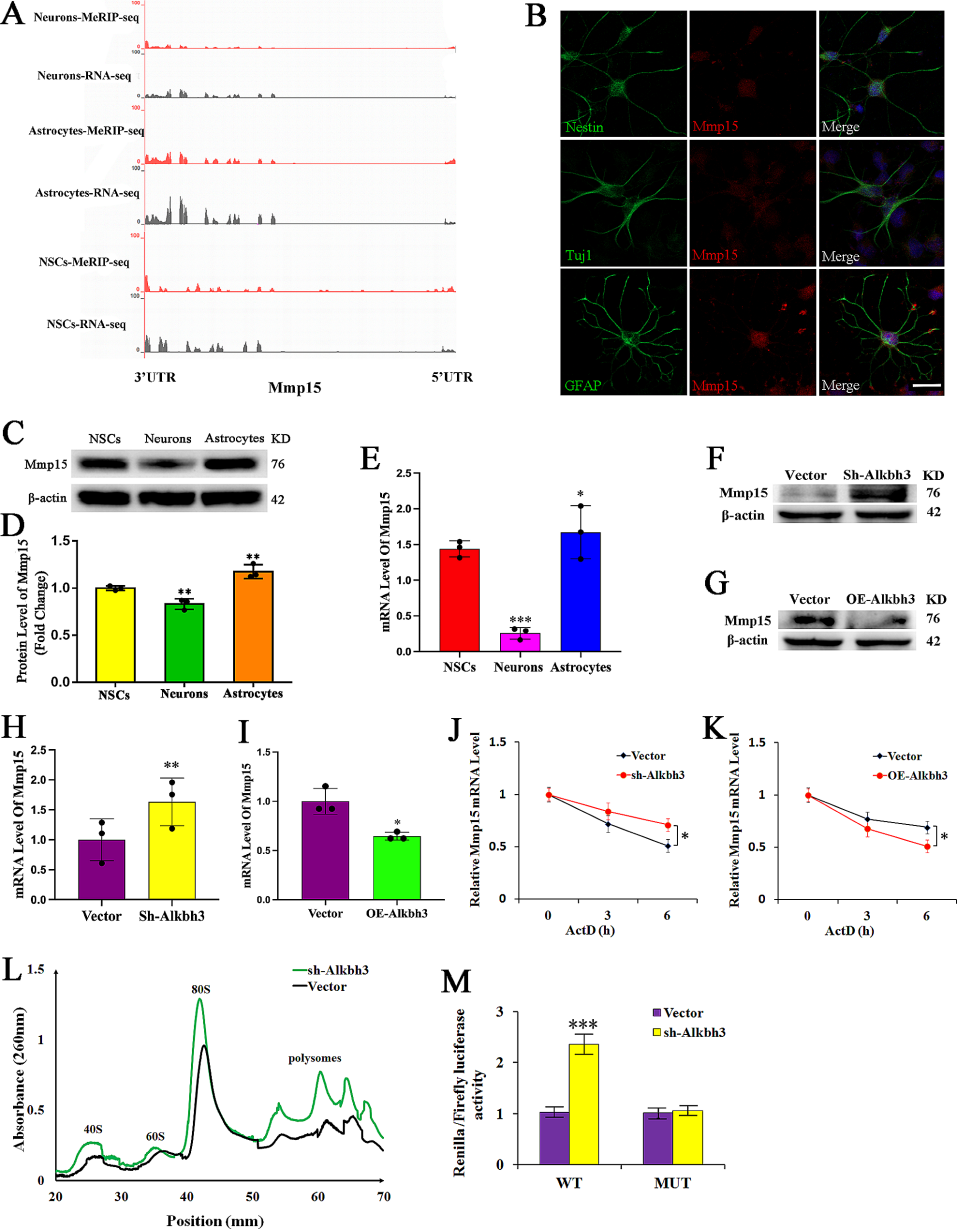
Alkbh3-mediated mRNA m^**1**^**A demethylation enhances Mmp15 stability and translation** (**A**) Integrative Genomics Viewer (IGV) displays the representative peaks of m^1^A-seq from NSCs, neurons and astrocytes. (**B-E**) Immunofluorescence staining, Western blot and RT-qPCR of Mmp15 expression in NSCs, neurons and astrocytes. Scale bar, 25 μm, *n* = 3, **P < 0.01, *P < 0.05 compared to NSCs. (**F-I**) The expression of Mmp15 protein and mRNA in NSCs with the depletion or overexpression of Alkbh3. *n* = 3, **P < 0.01, *P < 0.05 compared with vector. (**J**,** K**) Quantitative RT-PCR of Mmp15 mRNA in vector and Alkbh3-depleted or overexpressed cells treated with actinomycin D at the indicated times. *n* = 3, *P < 0.05 compared with vector. (**L**) Polysome profiling of Mmp15 mRNA in NSCs with or without Alkbh3 knockdown after sucrose gradient centrifugation. (**M**) Relative luciferase activity of WT or MUT Mmp15-3ʹUTR luciferase reporter in cells transfected with vector or Sh-Alkbh3 virus. *n* = 3, ***P < 0.001 compared with vector. Data are represented as the mean ± SEM. NSCs, neural stem cells; Tuj1, β-tubulin; GFAP, glial fibrillary acidic protein; Edu, 5-ethynyl-2′-deoxyuridine; WT, wildtype; MUT, mutanttype; Sh-Alkbh3, shRNA Alkbh3; OE-Alkbh3, overexpressing Alkbh3; ActD, actinomycin D; neurons, neuronal differentiation; astrocytes, astrocyte differentiation; n represents number of independent experiments

Comparison of m^1^A modification patterns in neuronal differentiation and NSCs confirmed many genes correlated with neurogenesis as m^1^A-modified targets including Mmp15 (Fig. 3K). Integrative Genomics Viewer (IGV) of Mmp15 gene showed a strong enrichment of m^1^A in 3′UTR and CDS in neuronal differentiation (Fig. 4A), indicating the crucial role of m^1^A modification of Mmp15 in regulating neurogenesis.

To assess whether m^1^A modification affects Mmp15 expression, we first detected Mmp15 expression in 3 types of cells. Unexpectedly, the findings from immunofluorescence, western blotting and RT-qPCR analyses showed that Mmp15 level was lowly expressed in neuronal differentiation and highly expressed in astrocyte differentiation compared with NSCs (Fig. 4B-E), suggesting that Mmp15 expression was strongly related with neurogenesis. To further investigate whether m^1^A modification of Mmp15 regulates its expression in neurogenesis, we depleted or overexpressed Alkbh3 to evaluate Mmp15 expression in NSCs and found that knockdown of Alkbh3 significantly increased expressions of Mmp15 protein and mRNA, while the expressions of Mmp15 protein and mRNA were significantly decreased by overexpression of Alkbh3 in NSCs (Fig. 4F-I), implying that Mmp15 is a potential target of Alkbh3-mediated m^1^A demethylation. Together, these data suggest that Alkbh3-mediated m^1^A demethylation enhances Mmp15 expression in neurogenesis.

We next asked if m^1^A demethylation regulates Mmp15 mRNA stability. RNA decay assay via actinomycin D–mediated transcription inhibition showed that knockdown Alkbh3 induced a slower degradation rate of Mmp15 mRNA, while the opposite effect was indicated on overexpression of Alkbh3 (Fig. 4J, K), indicating that Alkbh3-mediated m^1^A demethylation promotes Mmp15 expression via stabilizing Mmp15 mRNA. Thereafter, we further investigated whether m^1^A demethylation affects mRNA translation via polysome profiling and found that knockdown of Alkbh3 induced an increase of the polyribosome peaks (Fig. 4L), suggesting that m^1^A demethylation enhances Mmp15 mRNA translation in neurogenesis. In addition, we performed the Mmp15 3′-UTR luciferase reporter assay to explore the molecular mechanism underlying the enhance of m^1^A-mediated Mmp15 mRNA translation and found that the relative luciferase activity of construct containing Mmp15 3′UTR was obviously upregulated in Alkbh3 knockdown cells, while downregulated after mutation at all sites in Alkbh3 knockdown cells (Fig. 4M). These results indicate that Alkbh3 regulates Mmp15 post-transcriptionally via m^1^A demethylation-dependent mechanism.

### Mmp15 is sufficient and necessary for Alkbh3-induced neurogenesis

**Fig. 5:**
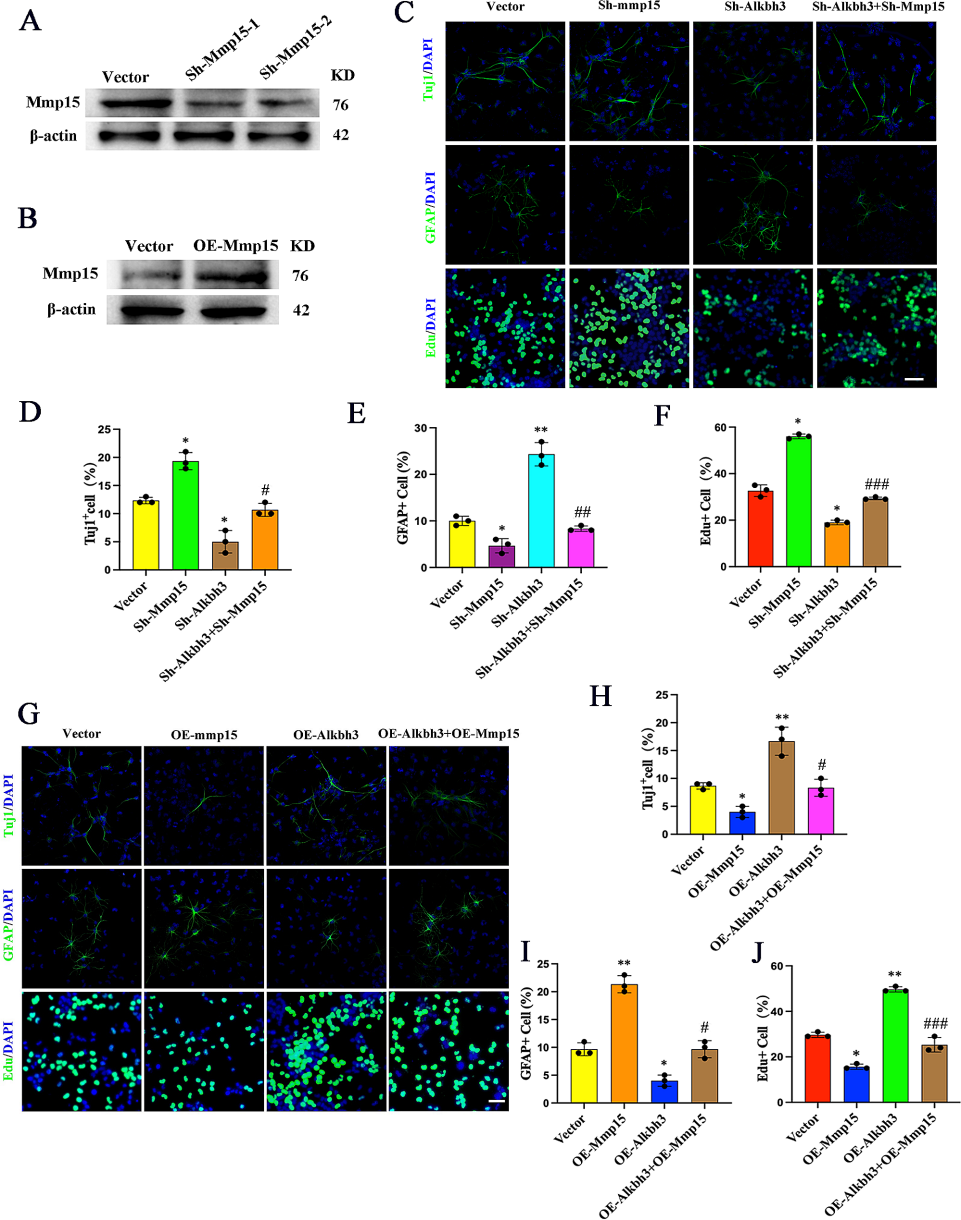
Mmp15 is essential for Alkbh3-induced neurogenesis (**A**,** B**) Western blotting of Mmp15 to validate the knockdown and overexpression efficiency in NSCs. (**C-F**) Immunofluorescence staining and quantification for Tuj1, GFAP, and EdU on NSCs treated with shRNA Alkbh3 or shRNA Mmp15. Scale bar, 50 μm. *n* = 3, *P < 0.05, **P < 0.01 compared with vector; #*P* < 0.05, ##*P* < 0.01, ###*P* < 0.001 compared with shRNA Alkbh3. (**G-J**) Immunofluorescence staining and quantification for Tuj1, GFAP, and EdU on NSCs treated with overexpressing Alkbh3 or overexpressing Mmp15. Scale bar, 50 μm. *n* = 3, *P < 0.05, **P < 0.01 compared with vector; #*P* < 0.05, ###*P* < 0.001 compared with OE-Alkbh3. Data are represented as the mean ± SEM. Sh-Alkbh3, shRNA Alkbh3; Sh-Mmp15, shRNA Mmp15; OE-Alkbh3, overexpressing Alkbh3; OE-Mmp15, overexpressing Mmp15; Tuj1, β-tubulin; GFAP, glial fibrillary acidic protein; n represents number of independent experiments

Since Alkbh3 regulates Mmp15 expression via mRNA m^1^A demethylation, we further analyzed the biological function of Mmp15 in the neurogenesis induced by the knockdown or overexpression of Alkbh3. Rescue experiments showed that Mmp15 depletion reversed the decreased neurogenesis induced by knockdown of Alkbh3 (Fig. 5A and C-F). In contrast, forced expression of Mmp15 restored the differentiation defect in Alkbh3-overexpressing NSCs (Fig. 5B and G-J). Taken together, our data demonstrate that Alkbh3-mediated m^1^A demethylation modulates neurogenesis via affecting Mmp15.

### Alkbh3 depletion produces decreased neurogenesis and cognitive impairment

**Fig. 6:**
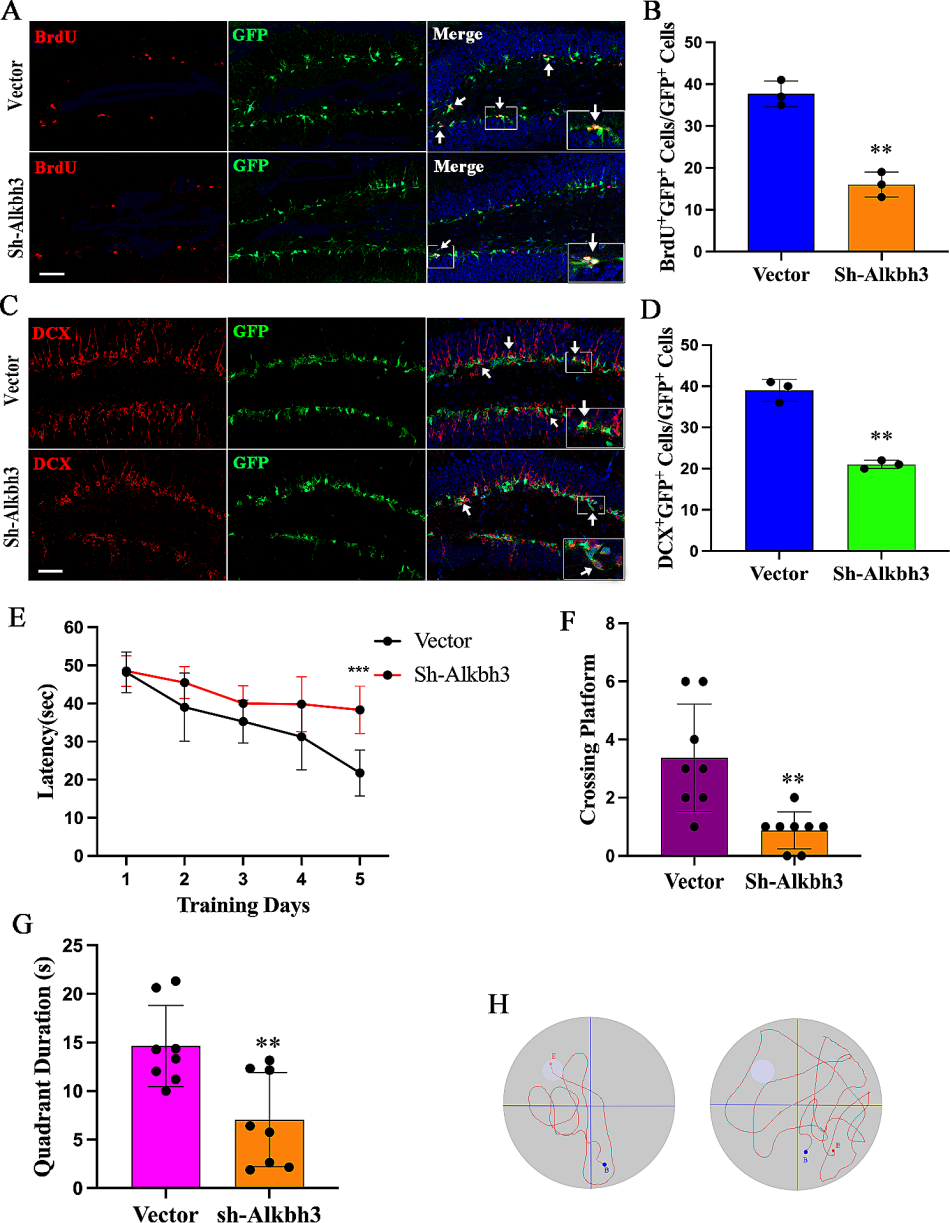
Alkbh3 depletion elicits decreased neurogenesis and cognitive deficit in mice **(A-D**) Representative images showing BrdU and DCX used for quantification in animals injected with Alkbh3 shRNA retrovirus. Scale bar, 50 μm. **P < 0.01 compared with vector. (**E**) The escape latency in the navigation test. ***P < 0.001 compared with vector. (**F**) The number of target crossings where the platform had been located in the probe test. **P < 0.01 compared with vector. (**G**) Quadrant time (%) in the probe test. **P < 0.01 compared with vector. (**H**) Representative swimming paths in each quadrant during the probe trial. *n* = 8 mice per group. Data are represented as the mean ± SEM. Sh-Alkbh3, shRNA Alkbh3; GFP, green fluorescent protein; BrdU, 5-Bromo-2′-deoxyuridine; DCX, doublecortin

To further validate our in vitro results, we then assessed whether Alkbh3 impacted hippocampal neurogenesis in vivo and retroviruses expressing Alkbh3 shRNA were injected into bilateral dentate gyrus of hippocampus. We observed that Alkbh3 knockdown significantly reduced neurogenesis (BrdU^+^/DCX^+^ cells) (Fig. 6A-D), confirming that Alkbh3 regulates adult hippocampal neurogenesis.

Given that decreased hippocampal neurogenesis induces cognitive impairment, we employed Morris water maze to assess spatial learning and memory of the mice and found that Alkbh3-deficency mice exhibited a significant cognitive decline during the last one session in the acquisition trial (Fig. 6E), suggesting an impaired spatial learning ability. In the probe trial, Alkbh3-deficency mice showed fewer number of crossing over the platform and spent less time in target quadrant compared to the control mice (Fig. 6F-H), implying spatial memory deficit.

## Discussion

m^1^A dynamic modification, demethylated by Alkbh3 and methylated by Trmt6/61a in mRNA, impacts RNA processing, stability and translational efficacy. Alkbh3 was initially found to repair DNA after alkylation damage and mostly located in the nucleus and cytoplasm [12]. Alkbh3-mediated m^1^A modification is greatly implicated in many biological processes including hematopoietic stem cells (HSCs) maintenance and differentiation, plant growth and development, ciliogenesis, apoptosis and oxidative damage [9, 11, 13, 14]. However, its physiological consequences in neurogenesis remain unclear. In this study, we find that Alkbh3 was enriched in neurons but scarcity in astrocytes compared to NSCs, while the opposite result was observed on m^1^A modification, which shows that Alkbh3-mediated m^1^A may modulate neuronal differentiation. Moreover, Alkbh3 depletion or overexpression severely affected the level of m^1^A methylation in NSCs and neurogenesis in vitro and in vivo. Thus, our results for the first time demonstrate the biological function of Alkbh3-mediated m^1^A modification during neurodevelopment, implying that the detection of Alkbh3/m^1^A is vital to enhance early estimation of neuronal genesis. However, additional work will be necessary to elucidate the mechanisms of biological function induced by the Alkbh3-mediated m^1^A in neuronal differentiation. In particular, research on the functions of m^1^A readers remained limited.

Alkbh3-mediated m^1^A modification is involved in multiple diseases related to carcinogenesis, nervous system diseases, cardiovascular diseases and pulmonary diseases [10, 15–17]. However, it remained to be elusive about the role of Alkbh3-mediated RNA demethylation in adult hippocampal neurogenesis and hippocampus-dependent spatial learning and memory. Herein, our data show that Alkbh3-deficency mice exhibits decreased hippocampal neurogenesis and a severe deficit in spatial learning and memory, suggesting targeting Alkbh3 regulation of neurogenesis as a potential therapeutic strategy for cognitive impairment. Our previous studies also demonstrate that internal mRNA m^7^G modification installed by Mettl1 and/or Mettl3-mediated m^6^A modification facilitate hippocampal neurogenesis [18, 19]. We infer whether these epigenetic modifications regulate hippocampal neurogenesis through modifying common gene, thereby improving hippocampal neurogenesis-related cognitive impairment. Therefore, the synergistic effects and precise mechanisms of the modifications need to be further investigated.

Mmp15 is a membrane-bound protease that degrades extracellular matrix and is a crucial mediator of basement membrane remodeling and cell invasiveness [20, 21]. Mmp15 is expressed in the cranial and caudal neuropores and implicated in endocardial cushion formation and submandibular gland morphogenesis in mouse development [22–25]. Moreover, Mmp15 regulates syncytial differentiation in primary human trophoblasts [26], placental development and function in early pregnancy [27], and their enhanced invasion in adipose-derived stromal cells [28], implying an crucial role of Mmp15 in neurodevelopment. However, the function and post-transcriptional regulation of Mmp15 in neurogenesis remains unclear. Our data reveal that Mmp15 is strongly enriched in astrocytes, while weakly expressed in neurons compared with NSCs, indicating that Mmp15 negatively regulates neuronal differentiation. More importantly, we identify that Alkbh3-mediated m^1^A demethylation promotes Mmp15 expression via catalyzing Mmp15 mRNA and enhancing its stability and translation in Alkbh3-induced neurogenesis. m^1^A modification has diversified effects on protein synthesis. m^1^A located in 5’UTR of mRNA is related with increased translation initiation and translation efficiency [29]. Conversely, m^1^A in the CDS of mRNA interferes with translation [30]. Strikingly, transcriptome analysis discovers that presence of m^1^A mainly enriched in 3’UTR correlates with increased protein expression of Mmp15 during neurogenesis. Thus, we provide solid evidence that Mmp15 was modified in 3’UTR and its expression was regulated by m^1^A modification in neurogenesis: (1) m^1^A-RIP-seq showed a significant enrichment of 3’UTR mRNA; (2) Alkbh3 regulated the m^1^A demethylation and Mmp15 expression via m^1^A-related enzyme activity. We cannot exclude the possibility that other mechanisms exist for the effects of m^1^A-modified transcripts, however these mechanisms remain yet unknown.

## Conclusion

In summary, our study reveals a vital role of Alkbh3-mediated m^1^A demethylation in adult hippocampal neurogenesis via enhancing Mmp15 mRNA stability and translation. Our findings further unveil that Alkbh3-deficency mice display reduced neurogenesis and cognitive decline. Our data also suggest targeting Alkbh3 in regulating neurogenesis as a novel therapeutic approach for neurogenesis-related cognitive impairment.

### Electronic supplementary material

Below is the link to the electronic supplementary material.

Supplementary Material 1

Supplementary Material 2

## Data Availability

All data will be provided upon availability and reasonable request.

## Abbreviations

m^1^A: N^1^-Methyladenosine
NSCs: neural stem cells
HSCs: hematopoietic stem cells
AHN: adult hippocampal neurogenesis
Alkbh3: Alpha-ketoglutarate-dependent dioxygenase 3
Mmp15: Matrix Metalloproteinases 15
GO: Gene ontology
IGV: Integrative Genomics Viewer
CDS: coding sequence
