# Supplementary material for: m1A demethylase Alkbh3 regulates neurogenesis through m1A demethylation of Mmp15 mRNA
Source: Cell Biosci. 2024 Jul 14;14:92. doi: 10.1186/s13578-024-01275-9 (PMC11246583; doi:10.1186/s13578-024-01275-9)
Supplement: Supplementary file 1 — Supplementary Material 1 [file 13578_2024_1275_MOESM1_ESM.docx]

Figure 1B


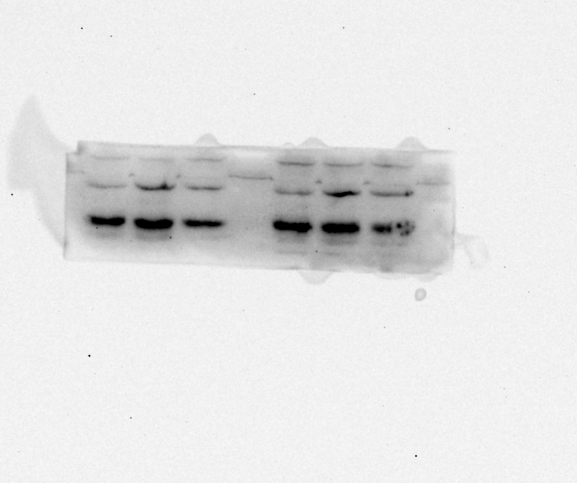

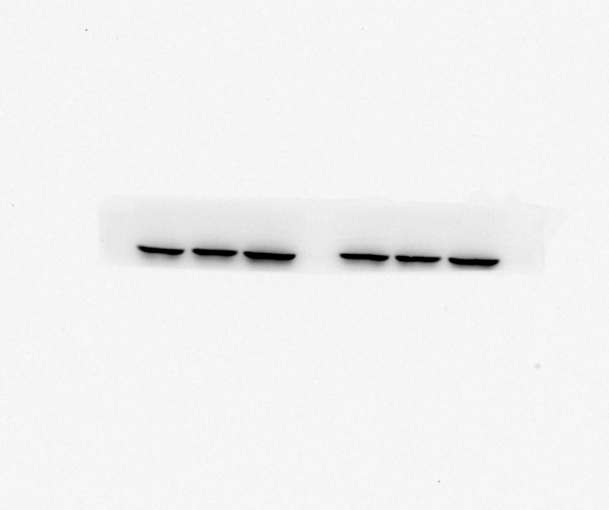


Alkbh3

β-actin

NSCs Neurons Astrocytes

NSCs Neurons Astrocytes

Figure 1E


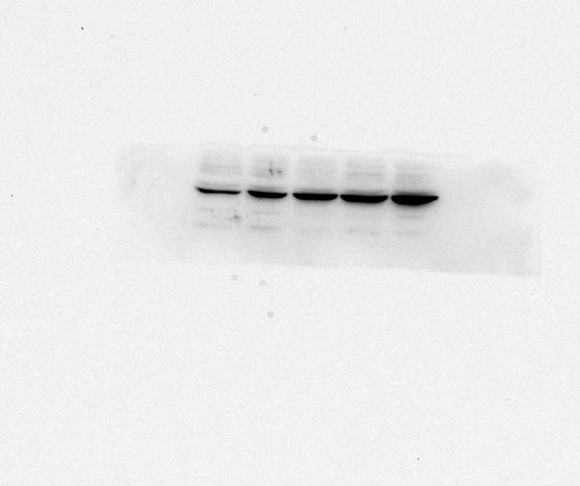

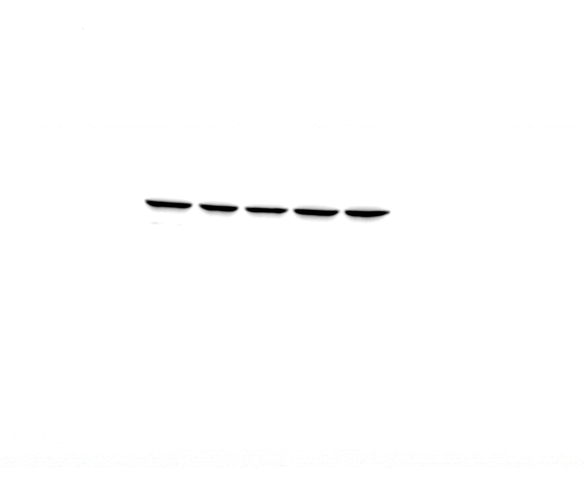


β-actin

Alkbh3


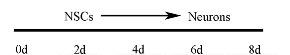


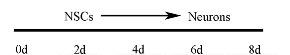


Figure 1G


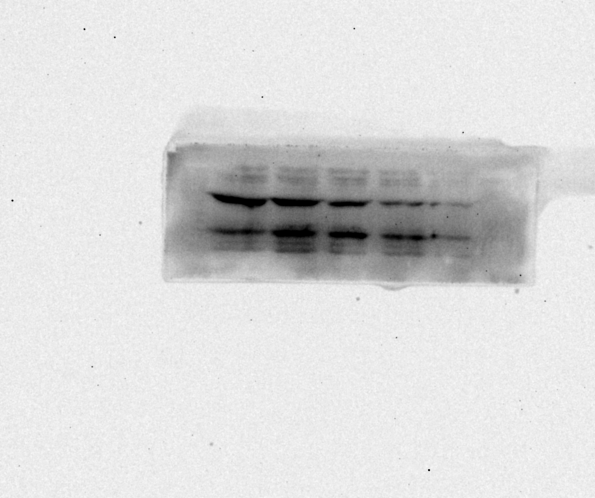

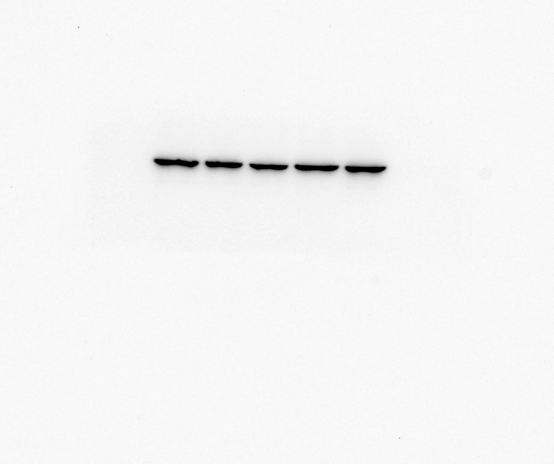


β-actin

Alkbh3

NSCs Astrocyte

NSCs Astrocyte

0 2d 4d 6d 8d

0 2d 4d 6d 8d

Figure 2A


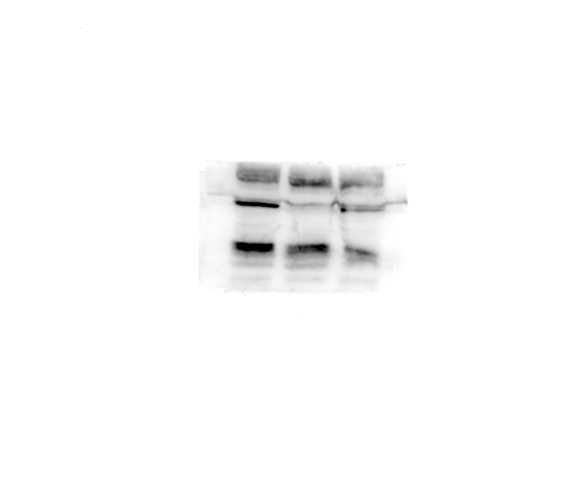

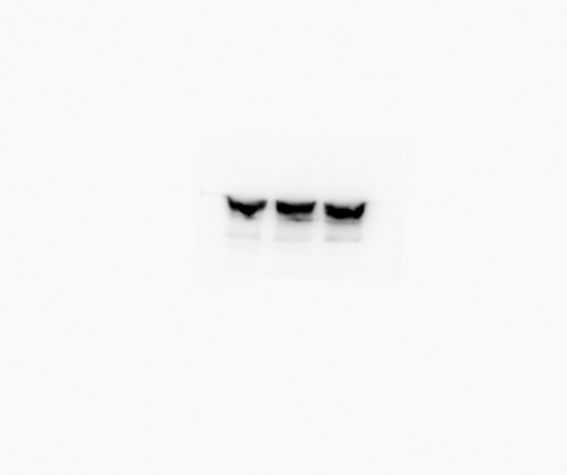


Vector sh-Alkbh-1 sh-Alkbh-2

β-actin

Alkbh3

Vector sh-Alkbh-1 sh-Alkbh-2

Figure 2G


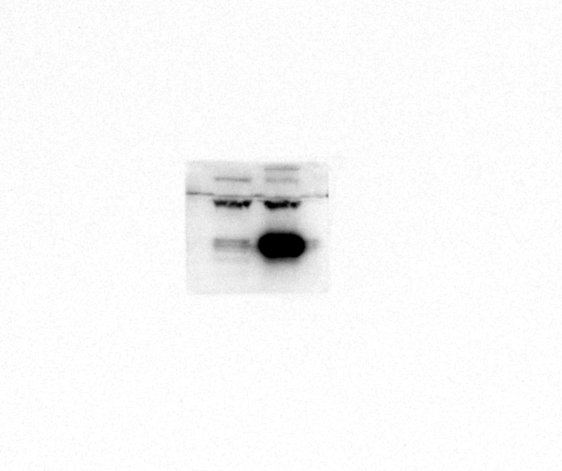

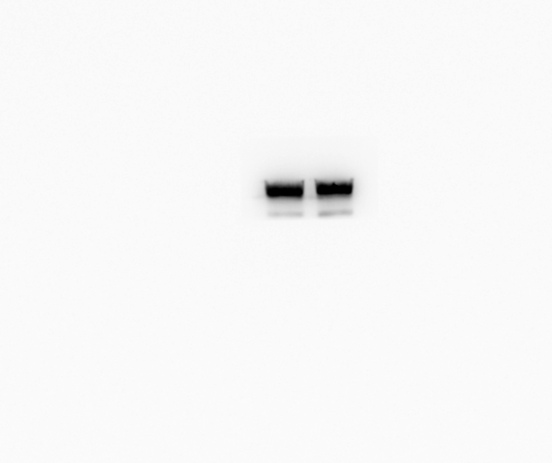


Vector OE-Alkbh3

Vector OE-Alkbh3

β-actin

Alkbh3

Figure 3A 3C 3E


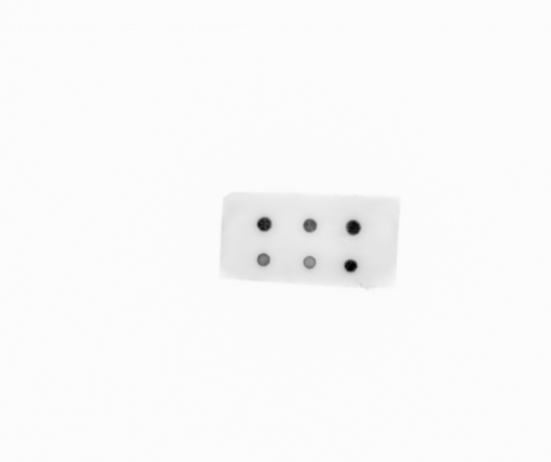

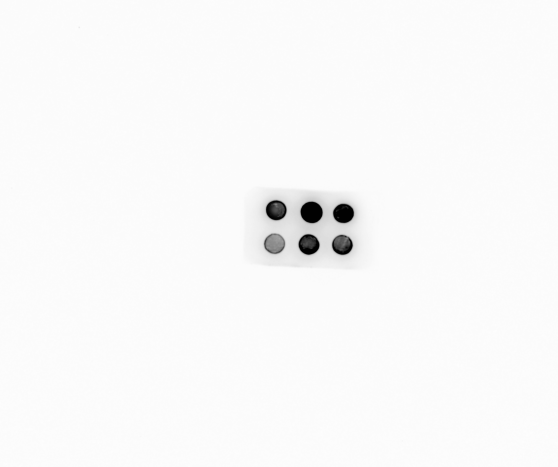


m^1^A

m^1^A

Vector sh-Alkbh-1 sh-Alkbh-2

NSCs Neurons Astrocytes


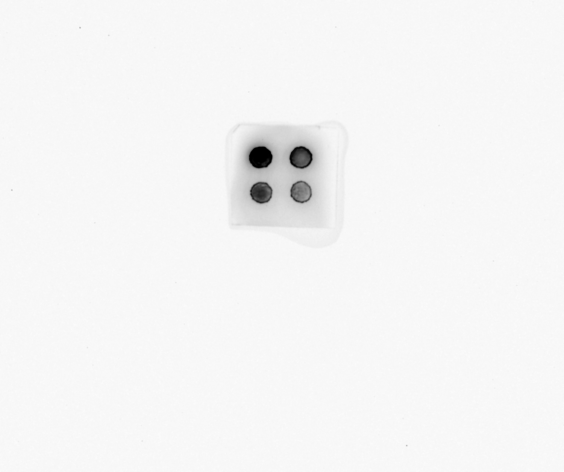


m^1^A

Vector OE-Alkbh3

Figure 4C


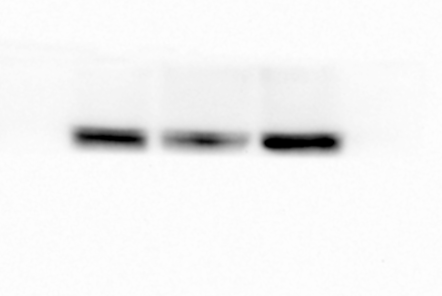

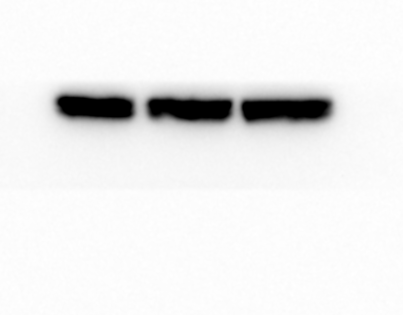


NSCs Neurons Astrocytes

Mmp15

β-actin

NSCs Neurons Astrocytes

Figure 4F


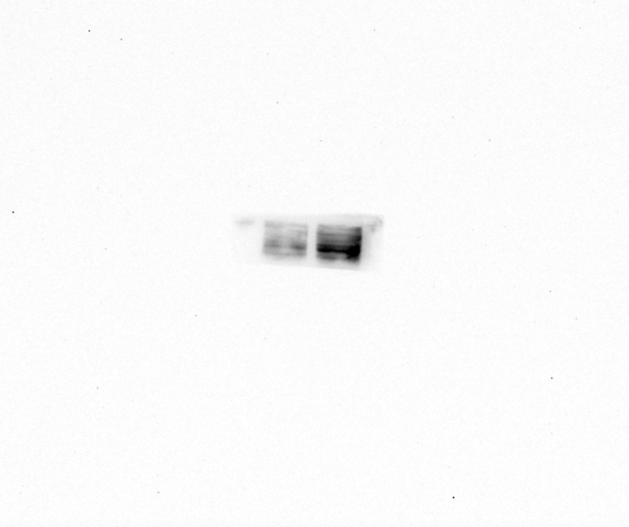

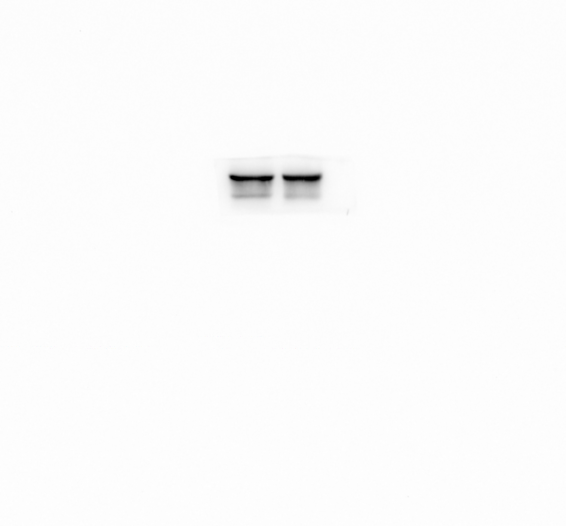


Vector Sh-Alkbh3

Vector Sh-Alkbh3

Mmp15

β-actin

Figure 4G


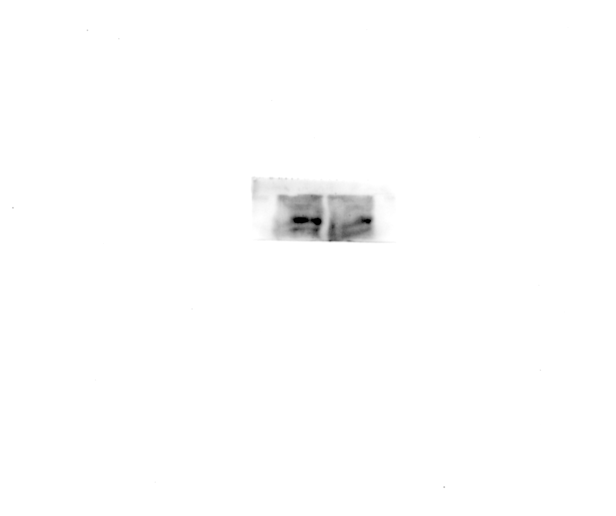

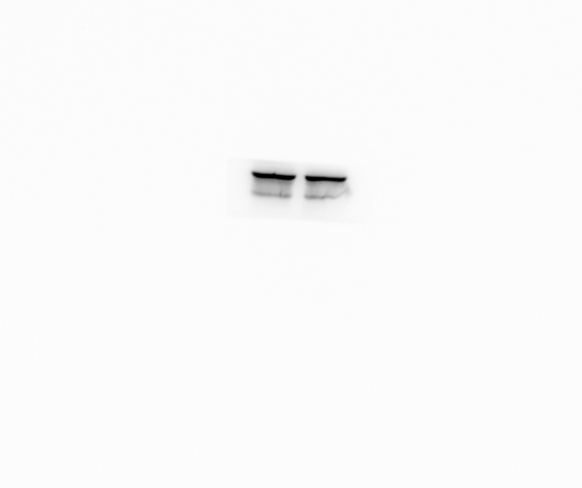


Vector OE-Alkbh3

Vector OE-Alkbh3

Mmp15

β-actin

Figure 5A


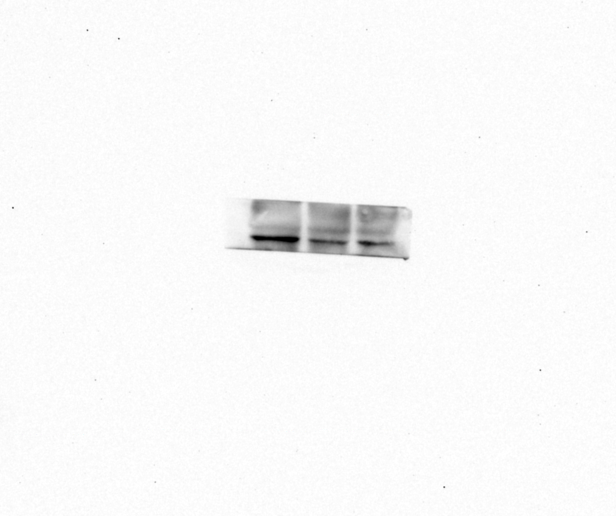

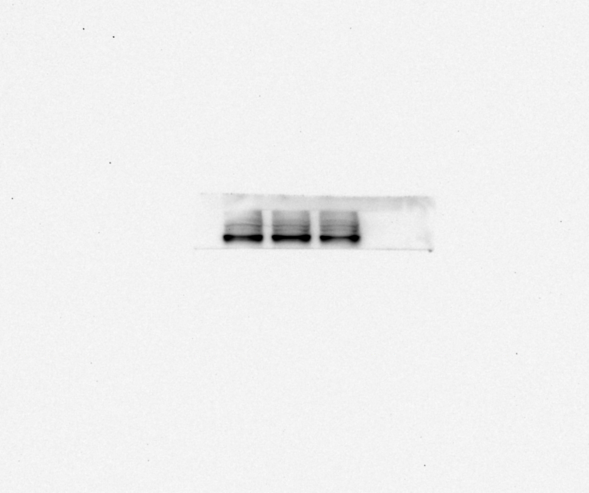


Vector sh-Mmp15-1 sh-Mmp15-2

Vector sh-Mmp15-1 sh-Mmp15-2

Mmp15

β-actin

Figure 5B


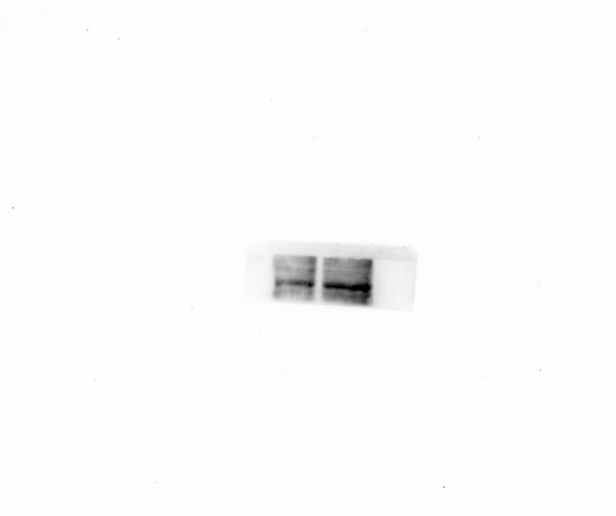

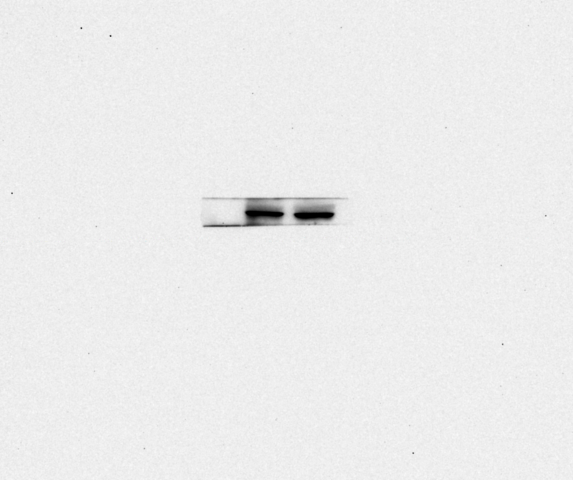


Vector OE-Mmp15

Vector OE-Mmp15

Mmp15

β-actin
