# Supplementary material for: m1A demethylase Alkbh3 regulates neurogenesis through m1A demethylation of Mmp15 mRNA
Source: Cell Biosci. 2024 Jul 14;14:92. doi: 10.1186/s13578-024-01275-9 (PMC11246583; doi:10.1186/s13578-024-01275-9)
Supplement: Supplementary file 2 — Supplementary Material 2 [file 13578_2024_1275_MOESM2_ESM.docx]

**Figure 1. The transcriptome of m^1^A-modified mRNA during astrocytic differentiation of NSCs.**

(**A, B**) Bar plot chart showing the significant KEGG and GO terms analysis for NSCs and differentiated astrocytes mRNAs containing m^1^A modification. (**C**) Motif searching identified the consensus conserved sequence. (**D**) Volcano plot of significantly altered mRNA m^1^A peaks in NSCs compared to astrocytes. NSCs, neural stem cells. astrocytes, astrocyte differentiation.

**
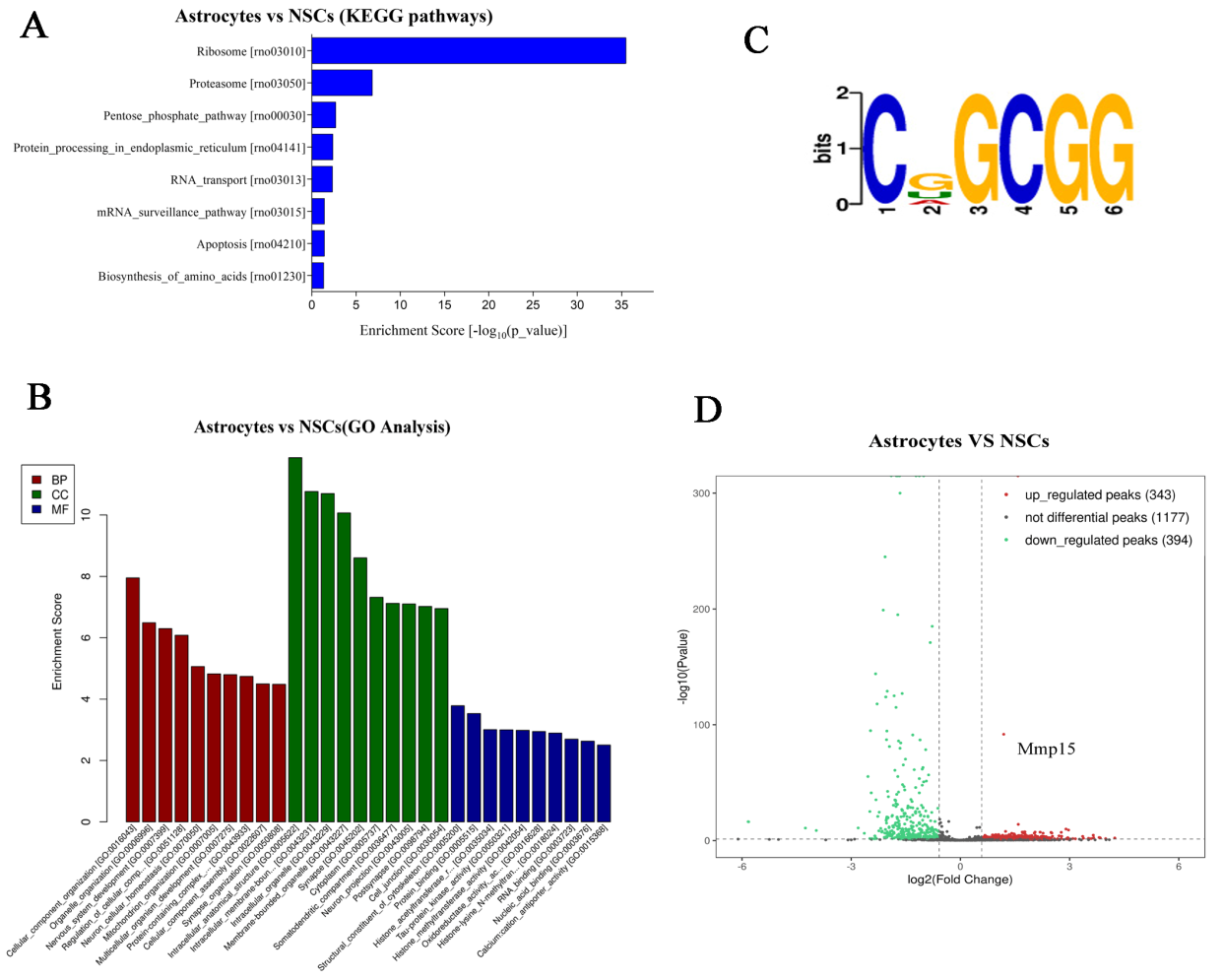
**
